# Effect of bucladesine calcium combined with metoprolol on heart rate variability and cardiac function prognosis in heart failure patients with chronic arrhythmias

**DOI:** 10.3389/fcvm.2026.1740664

**Published:** 2026-05-04

**Authors:** Mengna Sun, Beibei Fan, Ming Lin, Kun Yan, Chao Yang, Zhao Gao

**Affiliations:** 1 Department of Cardiology, Xi'an International Medical Center Hospital, Xi'an, Shaanxi, China; 2 Department of Nephrology, Shaanxi Provincial Crops Hospital of Chinese People's Armed Police Force, Xi'an, Shaanxi, China; 3 Department of Pharmacy, Shaanxi Provincial Crops Hospital of Chinese People’s Armed Police Force, Xi'an, Shaanxi, China

**Keywords:** bucladesine, cardiac function, chronic arrhythmia, heart failure, heart rate variability, metoprolol

## Abstract

**Background:**

Heart failure (HF) with chronic arrhythmias is associated with autonomic dysfunction and poor outcomes. Heart rate variability (HRV) reflects autonomic dysfunction and predicts outcomes in HF. This study investigated whether adding bucladesine calcium to metoprolol improves HRV and cardiac function compared with metoprolol alone.

**Methods:**

This single-center retrospective observational study included 87 patients with HF and chronic arrhythmia treated between January 2023 and April 2024. Patients received metoprolol tartrate alone (control group, *n* = 41) or bucladesine calcium combined with metoprolol (observation group, *n* = 46) for 12 weeks. The primary outcome was change from baseline in HRV time-domain parameters. Between-group differences were compared with 95% confidence intervals. Exploratory sensitivity and multivariable-adjusted analyses were performed.

**Results:**

Baseline characteristics were comparable between groups. The combination group showed significantly greater improvements in ΔSDNN [mean difference 13.63 ms, 95% CI (4.17, 23.09), *P* = 0.005] and ΔRMSSD [8.94 ms, (2.80, 15.08), *P* = 0.005]. ΔLF/HF showed a borderline difference (*P* = 0.043). ΔLVEF was significantly greater [4.86%, (1.31, 8.41), *P* = 0.008], as was ΔCO (*P* = 0.019), while ΔLVEDd did not differ significantly (*P* = 0.395). The overall response rate was numerically higher (89.1% vs. 78.0%) but not statistically significant (*P* = 0.266). Adverse event rates were comparable. After multivariable adjustment, ΔSDNN (*P* = 0.037) and ΔRMSSD (*P* = 0.042) remained significant, while ΔLVEF became borderline (*P* = 0.062).

**Conclusion:**

Adding bucladesine calcium to metoprolol was associated with greater improvements in HRV time-domain parameters and LVEF, though effects were attenuated after covariate adjustment.

## Introduction

Heart failure (HF) remains a major global health challenge with increasing prevalence. The lifetime risk of developing HF has increased to 24%, meaning approximately one in four individuals will develop this condition during their lifetime (1). Chronic arrhythmias, particularly atrial fibrillation (AF) and ventricular arrhythmias, are present in up to 50% of HF patients and significantly contribute to disease progression and adverse outcomes (2). AF and HF often coexist and can precipitate one another, which can increase mortality risk, stroke incidence, and hospitalizations (3, 4). The complex interaction between cardiac dysfunction, neurohormonal activation, and autonomic dysregulation in these patients necessitates therapeutic approaches that address multiple pathophysiological mechanisms simultaneously (4).

Heart rate variability (HRV) has emerged as a crucial noninvasive marker for assessing autonomic nervous system function and predicting outcomes in HF (5). HRV reflects the dynamic interplay between sympathetic and parasympathetic influences on cardiac rhythm, and reduced HRV parameters are strongly associated with increased mortality and adverse cardiac events (5). A low frequency-to-high frequency ratio, an index of autonomic balance, has been shown to predict the development of HF in elderly cohorts, with the addition of HRV parameters to traditional cardiovascular risk factors improving predictive performance by 6.1% (6). Recent studies have established reference values for HRV markers in HF populations and confirmed that HRV values outside the reference range are significantly associated with mortality, underscoring the clinical importance of HRV assessment in risk stratification (5, 7). Therapeutic interventions that improve HRV may therefore offer prognostic benefits beyond traditional measures of cardiac function by addressing the fundamental autonomic imbalance that characterizes HF.

Metoprolol, a selective beta-1 adrenergic receptor antagonist, remains a cornerstone of guideline-directed medical therapy for HF with reduced ejection fraction (8). The 2024 ACC Expert Consensus Decision Pathway emphasizes the importance of beta-blockers as one of the “four pillars” of treatment, alongside angiotensin receptor-neprilysin inhibitors, mineralocorticoid receptor antagonists, and sodium-glucose cotransporter-2 inhibitors (9). Evidence-based beta-blocker use, including carvedilol, bisoprolol, and sustained-release metoprolol succinate, is associated with lower HF readmission rates and mortality in real-world Medicare populations (10). Metoprolol exerts its beneficial effects through multiple mechanisms, including reduction of heart rate and blood pressure, attenuation of pathological neurohormonal activation, improvement of left ventricular function, and restoration of downregulated beta-adrenergic receptor signaling pathways. Bucladesine calcium, a cell-permeable cyclic adenosine monophosphate analog, functions as a protein kinase A activator and phosphodiesterase inhibitor (11, 12). By mimicking endogenous cAMP and enhancing intracellular signaling pathways, bucladesine calcium has potential to improve myocardial contractility and modulate cardiac function through mechanisms that may complement beta-blocker therapy.

Despite the benefits of metoprolol monotherapy and advances in guideline-directed medical therapy, many patients with HF and chronic arrhythmias continue to experience suboptimal outcomes, with persistent symptoms, recurrent hospitalizations, and elevated mortality risk. This study aims to evaluate whether the combination of bucladesine calcium and metoprolol provides superior benefits compared to metoprolol alone in improving HRV and cardiac function in patients with HF and chronic arrhythmias.

## Materials and methods

### Study design

This retrospective, observational cohort study reviewed medical records of patients with HF complicated by chronic arrhythmia treated at Xi'an International Medical Center Hospital between January 2023 and April 2024. This study was reported in accordance with the Strengthening the Reporting of Observational Studies in Epidemiology (STROBE) statement. The study protocol was approved by the institutional ethics committee (Approval No. 20240124), and the requirement for informed consent was waived due to the retrospective nature of the study. This study was conducted in accordance with the Declaration of Helsinki.

Patients were assigned to receive either metoprolol alone or metoprolol plus bucladesine calcium based on the attending physician's clinical decision at admission, guided the perceived need for additional inotropic support. The primary outcome was the change from baseline (Δ) in HRV time-domain parameters (SDNN and RMSSD) at 12 weeks. Secondary outcomes included changes in LF/HF ratio, cardiac function parameterss, clinical efficacy, and safety.

### Study population

HF was diagnosed according to the 2021 European Society of Cardiology (ESC) guidelines, requiring the presence of symptoms and/or signs of HF combined with objective evidence of cardiac dysfunction on echocardiography (13). Serum NT-proBNP was used to support the diagnosis, with a threshold of ≥125 pg/mL for chronic HF. All patients were classified by HF phenotype based on baseline left ventricular ejection fraction (LVEF): HF with reduced ejection fraction (HFrEF, LVEF <40%), HF with mildly reduced ejection fraction (HFmrEF, LVEF 40%–49%), or HF with preserved ejection fraction (HFpEF, LVEF ≥50%).

Chronic arrhythmia was defined as any documented arrhythmia persisting or recurring for ≥3 months before enrollment. AF was subclassified as paroxysmal, persistent, long-standing persistent, or permanent according to the American College of Cardiology/American Heart Association/American College of Chest Physicians/Heart Rhythm Society guidelines (14). Ventricular arrhythmias were categorized as frequent premature ventricular complexes (PVCs; ≥1,000/24 h on Holter), non-sustained ventricular tachycardia (NSVT), or both. Arrhythmia burden was quantified from 24-h Holter reports as the percentage of time in AF or the number of PVCs per 24 h.

A total of 87 patients were included in this analysis. Patients were divided into two groups based on their treatment regimen: the control group (*n* = 41) received metoprolol tartrate alone, while the observation group (*n* = 46) received bucladesine calcium combined with metoprolol tartrate.

Patients were eligible for inclusion if they met the following criteria: confirmed HF diagnosis and chronic arrhythmia per the criteria above; classification according to New York Heart Association (NYHA) functional class (15); clinically stable for ≥4 weeks since the last decompensation episode; age between 18 and 80 years, and complete medical records with baseline and follow-up data. Patients were excluded if they presented with acutely decompensated HF; sustained ventricular tachycardia or ventricular fibrillation requiring emergency intervention; significant hepatic or renal dysfunction; or contraindications to the study medications.

Background guideline-directed medical therapy at enrollment was documented for all patients and is summarized in Table 1. Recorded medications included angiotensin-converting enzyme inhibitors (ACEi) or angiotensin receptor blockers (ARB), angiotensin receptor–neprilysin inhibitors (ARNI), mineralocorticoid receptor antagonists (MRA), sodium-glucose cotransporter-2 inhibitors (SGLT2i), diuretics, digoxin, and anticoagulants. Antiarrhythmic drug use and prior ablation history were also recorded.

**Table 1:** Comparison of baseline characteristics between the two groups.

Characteristics	Observation group (*n* = 46)	Control group (*n* = 41)	χ*^2^/t*	*P*
Demographics				
Sex, *n* (%)				
Male	26 (56.52)	24 (58.54)	0.036	0.850
Female	20 (43.48)	17 (41.46)		
Age (years)	52.16 ± 4.88	51.23 ± 4.60	0.912	0.365
Disease duration (years)	2.40 ± 0.25	2.35 ± 0.29	0.864	0.390
BMI (kg/m^2^)	23.91 ± 2.76	24.08 ± 3.11	0.270	0.788
Vital signs				
SBP (mmHg)	122.43 ± 15.44	124.12 ± 15.04	0.515	0.608
DBP (mmHg)	73.26 ± 9.53	73.93 ± 9.98	0.318	0.751
Resting heart rate, bpm	82.50 ± 12.44	83.11 ± 11.94	0.233	0.816
NYHA classification, *n* (%)				
Class II	20 (43.48)	22 (53.66)	2.160	0.340
Class III	18 (39.13)	10 (24.39)		
Class IV	8 (17.39)	9 (21.95)		
HF phenotype, *n* (%)^a^				
HFrEF	25 (54.35)	25 (60.98)	0.166	0.684
HFmrEF	21 (45.65)	15 (36.59)		
HFpEF	0 (0.00)	1 (2.44)		
Etiology of HF, *n* (%)				
CHD	15 (32.61)	12 (29.27)	4.722	0.193
AF	9 (19.57)	14 (34.15)		
Hypertension	17 (36.96)	8 (19.51)		
DCM	5 (10.87)	7 (17.07)		
Types of arrhythmias, *n* (%)				
Atrial	24 (52.17)	22 (53.66)	0.019	0.890
Ventricular	22 (47.83)	19 (46.34)		
AF subtypes				
Paroxysmal AF	10 (41.7)	9 (40.9)		
Persistent AF	8 (33.3)	7 (31.8)		
Long-standing persistent/Permanent AF	6 (25.0)	6 (27.3)		
NSVT	10 (45.5)	6 (31.6)		
Frequent PVCs	8 (36.4)	11 (57.9)		
PVCs + NSVT	4 (18.2)	2 (10.5)		
Laboratory values				
NT-proBNP, pg/ml	1,594.61 ± 1,001.62	1,594.29 ± 879.67	0.298	0.769
eGFR, ml/min/1.73 m^2^	78.50 ± 18.40	76.80 ± 19.74	0.416	0.679
Hemoglobin, g/L	132.50 ± 16.99	131.20 ± 17.72	0.349	0.728
Comorbidities, *n* (%)				
Hypertension	25 (54.3)	20 (48.8)	0.269	0.604
Diabetes mellitus	15 (32.6)	13 (31.7)	0.008	0.928
CKD	10 (21.7)	12 (29.3)	0.651	0.430
Background GDMT, *n* (%)				
ACEi/ARB	29 (63.0)	28 (68.3)	0.265	0.607
ARNI	8 (17.4)	7 (17.1)	0.000	1.000
MRA	15 (32.6)	19 (46.3)	1.717	0.190
SGLT2i	6 (13.0)	6 (14.6)	0.000	1.000
Diuretics	35 (76.1)	33 (80.5)	0.246	0.620
Metoprolol				
Final achieved dose, mg/dose bid	19.84 ± 5.64	21.19 ± 4.82	0.952	0.297

Values are mean ± SD or *n* (%). AF subtype percentages calculated among atrial arrhythmia patients; ventricular subtype percentages among ventricular arrhythmia patients. CHD, coronary heart disease; AF, atrial fibrillation; DCM, dilated cardiomyopathy; PVC, premature ventricular complex; NSVT, non-sustained ventricular tachycardia; GDMT, guideline-directed medical therapy.

a HFmrEF and HFpEF combined for testing (low expected frequency).

### Treatment protocol

Both groups received standard HF management, comprising cardiac support, oxygen therapy as needed, correction of water-electrolyte imbalances, diuretic therapy, dietary counseling, and lifestyle guidance.

Patients in the control group were treated with metoprolol tartrate (AstraZeneca Pharmaceutical Co., Ltd.), starting at an initial dose of 6.25 mg bid, with gradual titration every 2–4 weeks to a target dose of 25 mg bid in accordance with the Chinese Guidelines for the Diagnosis and Treatment of Heart Failure (16). If the target dose was not tolerated, the maximum tolerated dose was used for maintenance (with a resting heart rate maintained above 55 bpm).

Patients in the observation group received bucladesine calcium in combination with metoprolol. The administration of metoprolol tartrate followed the same protocol as described for the control group. Additionally, bucladesine calcium (Shanghai No.1 Biochemical & Pharmaceutical Co., Ltd.) was administered at a dose of 40 mg per session, dissolved in 100 ml of 5% glucose solution, and given via intravenous infusion once daily. Treatment was initiated during hospitalization (median 2 weeks) for clinical stabilization and safety monitoring; thereafter, patients transitioned to a day-hospital or outpatient infusion center for the remainder of the 12-week course. Both groups continued treatment for 12 weeks.

### Outcome measures

All outcome data were retrospectively collected from patients' medical records, laboratory reports, and imaging archives.

(1) HRV parameters were obtained from 24-h Holter monitoring performed at baseline and at 12 weeks. HRV analysis followed the standards established by the Task Force of the European Society of Cardiology and the North American Society of Pacing and Electrophysiology. Only normal-to-normal (NN) intervals during sinus rhythm were included. Ectopic beats and AF segments were excluded from analysis. HRV indices included: standard deviation of all normal-to-normal RR intervals (SDNN, ms), the ratio of low frequency power (0.04–0.15 Hz) to high frequency power (0.15–0.40 Hz) (LF/HF ratio), and root mean square of successive differences between adjacent normal RR intervals (RMSSD, ms).
(2) Cardiac function parameters were extracted from transthoracic echocardiography performed at baseline and at 12 weeks. LVEF was measured using the modified Simpson's biplane method; cardiac output (CO), calculated as stroke volume × heart rate); left ventricular end-diastolic diameter (LVEDd) was measured in the parasternal long-axis view.
(3) Clinical efficacy and safety were assessed at 12 weeks based on changes in NYHA functional classification. Marked improvement was defined as improvement by ≥2 NYHA class; moderate improvement as 1 class; and no improvement as no change or worsening. The overall response rate was the proportion of patients achieving marked or moderate improvement.
(4) Rehospitalization rate was defined was defined as unplanned readmission for worsening HF or arrhythmia during the treatment and follow-up period. All-cause mortality and adverse events were recorded. Post-treatment resting heart rate was documented as a beta-blocker tolerability measure.

### Statistical analysis

Statistical analyses were performed using SPSS version 26.0 (IBM Corp.) with supplementary analyses in Python 3.11 (SciPy 1.11). All tests were two-tailed, *P* < 0.05 was considered significant. Continuous variables (mean ± SD) were compared using *t*-test or Mann–Whitney *U*-test according to the Shapiro–Wilk test; categorical variables (*n*, %) were compared using the χ^2^ test or Fisher's exact test.

The primary analysis compared between-group differences in Δ values (post-treatment minus baseline) using the independent-samples *t*-test or Mann–Whitney *U*-test, reporting the mean difference with 95% confidence intervals (CI). The Holm–Bonferroni correction was applied to the two co-primary endpoints (ΔSDNN and ΔRMSSD) to control for multiple testing. Secondary endpoints were analyzed without multiplicity adjustment and are reported as exploratory.

Sensitivity analyses were performed: (1) exclusion of patients with AF as the primary HF etiology; and (2) multivariable linear regression adjusting for age, sex, NYHA class, HF phenotype, baseline LVEF, NT-proBNP, AF etiology, and number of background GDMT agents. Given the limited sample size, these adjusted analyses were exploratory. Missing data were handled by complete-case analysis.

## Results

### Patient selection

**Figure 1:**
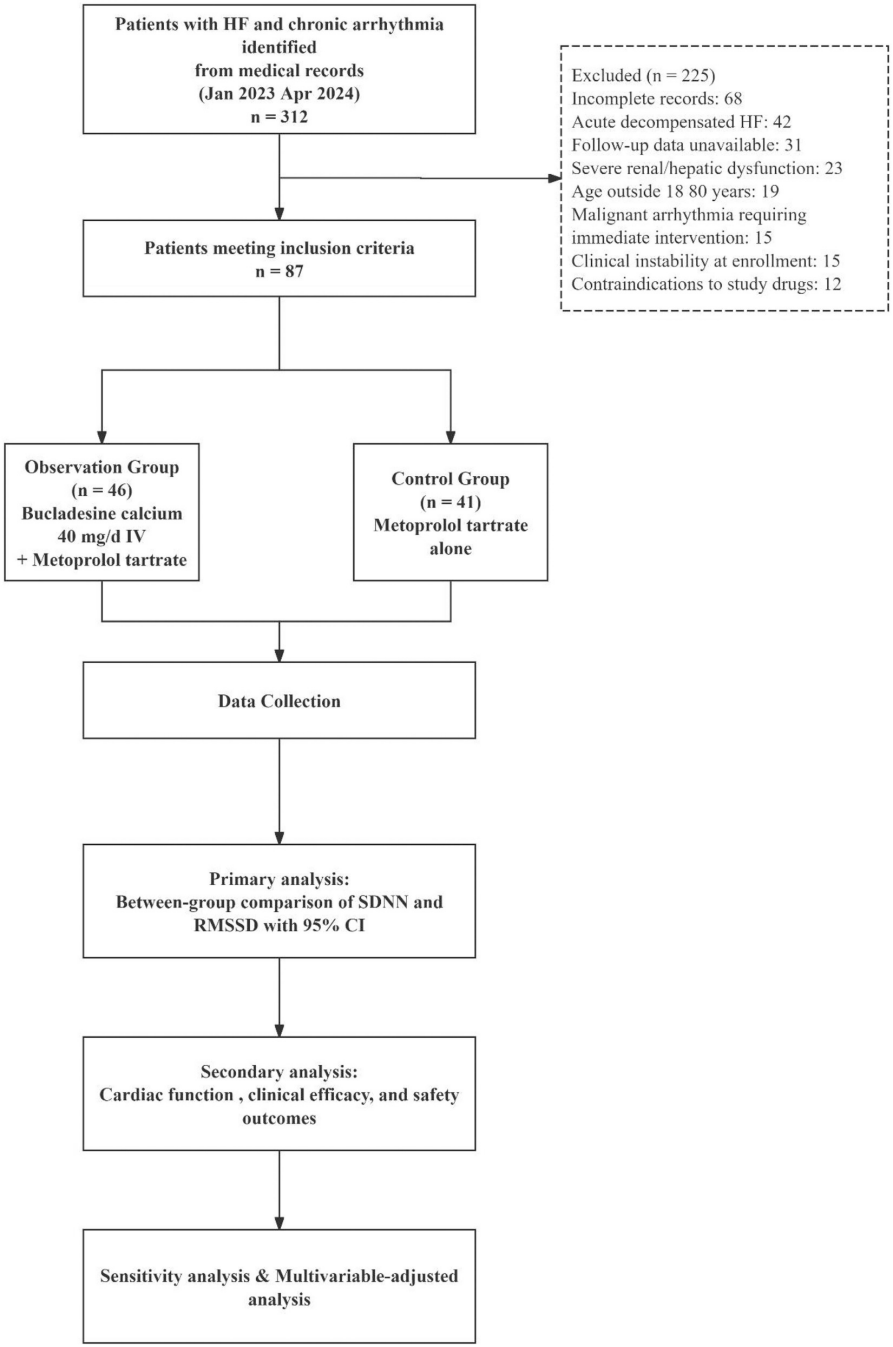
Patient selection flow diagram. HF, heart failure; HFrEF, heart failure with reduced ejection fraction (LVEF <40%); HFmrEF, heart failure with mildly reduced ejection fraction (LVEF 40 49%); HFpEF, heart failure with preserved ejection fraction (LVEF 50%); AF, atrial fibrillation; GDMT, guideline-directed medical therapy; IV, intravenous; CI, confidence interval.

A total of 312 patients with heart failure and chronic arrhythmia treated at our institution between January 2023 and April 2024 were identified through medical record review. After applying the inclusion and exclusion criteria, 87 patients were included in the final analysis: 46 in the observation group (bucladesine calcium combined with metoprolol) and 41 in the control group (metoprolol alone). The patient selection process is summarized in Figure 1.

### Baseline characteristics

The baseline characteristics of the two groups are presented in Table 1. No statistically significant differences were observed between the observation group and control group in terms of gender distribution (*P* = 0.850), mean age (*P* = 0.365), disease duration (*P* = 0.390), or BMI (*P* = 0.788). The distribution of NYHA functional classes was comparable between groups (*P* = 0.340). The majority of patients had HFrEF (observation 54.3%, control 61.0%) or HFmrEF (observation 45.7%, control 36.6%), with no significant difference in HF phenotype distribution between groups (*P* = 0.684).

Regarding the etiology of HF, both groups showed similar distributions of underlying causes including coronary heart disease, AF, hypertension, and dilated cardiomyopathy (*P* = 0.193). Among atrial arrhythmias, paroxysmal and persistent AF were the most common subtypes in both groups. Frequent PVCs and non-sustained ventricular tachycardia accounted for the majority of ventricular arrhythmias. Baseline laboratory values, including NT-proBNP, serum creatinine, eGFR, and hemoglobin, were comparable between groups (*P* all >0.05). Baseline echocardiographic parameters, including LVEF, CO, LVEDd, and E/e′ ratio, showed no significant between-group differences (*P* all >0.05).

Background guideline-directed medical therapy was similar between groups: ACEi/ARB, ARNI, MRA, SGLT2i, and diuretics, with no significant differences (all *P* > 0.05). The final achieved metoprolol dose was also comparable between groups (*P* = 0.297).

These findings indicate that the baseline characteristics were well-balanced between the observation and control groups, providing a valid basis for subsequent comparisons.

### Primary outcome: heart rate variability

**Figure 2:**
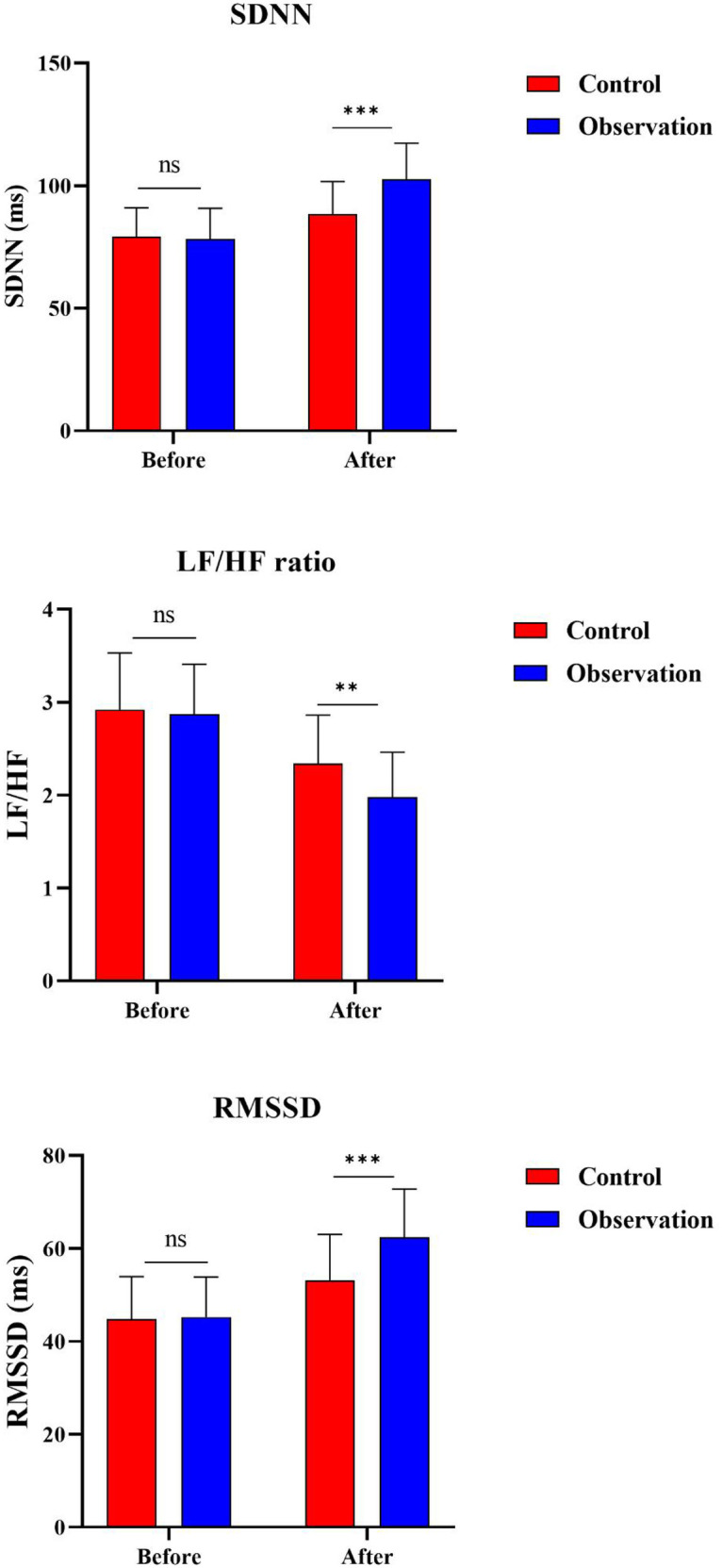
Changes in HRV parameters before and after treatment. **(A)** SDNN (standard deviation of all normal RR intervals); **(B)** RMSSD (root mean square of successive differences); **(C)** LF/HF ratio (low-frequency to high-frequency power ratio). ** *P* < 0.001.

Figure 2 demonstrates the comparison of HRV parameters between the two groups before and after treatment. At baseline, the observation group and control group showed comparable HRV indices with no statistically significant differences in SDNN (*P* = 0.751), LF/HF ratio (*P* = 0.681), or RMSSD (*P* = 0.841).

Following 12 weeks of treatment, both groups exhibited improvements in HRV parameters. The change from baseline in SDNN was significantly greater in the observation group than in the control group [ΔSDNN: 22.23 ± 22.79 vs. 8.60 ± 21.56 ms; mean difference 13.63, 95% CI (4.17–23.09), *P* = 0.005]. Similarly, the change in RMSSD was significantly greater in the observation group [ΔRMSSD: 19.22 ± 13.77 vs. 10.28 ± 14.89 ms; mean difference 8.94, 95% CI (2.80–15.08), *P* = 0.005]. The change in LF/HF ratio showed a modest between-group difference [ΔLF/HF: −0.88 ± 0.66 vs. −0.55 ± 0.84; mean difference −0.33, 95% CI (−0.66 to −0.01), *P* = 0.043]. The post-treatment values and change-from-baseline results for all HRV parameters are presented in Table 2.

**Table 2:** Comparison of HRV parameters before and after treatment.

Parameter	Observation group (*n* = 46)	Control group (*n* = 41)	Mean difference (95% CI)	*P*
SDNN, ms				
Baseline	80.35 ± 13.24	79.82 ± 12.76	0.53 (−5.02–6.08)	0.850
Post-treatment	102.58 ± 14.89	88.42 ± 13.42	14.16 (8.12–20.20)	<0.001
Δ Change from baseline	22.23 ± 22.79	8.60 ± 21.56	13.63 (4.17–23.09)	0.005
LF/HF ratio				
Baseline	2.86 ± 0.56	2.89 ± 0.53	−0.03 (−0.26–0.20)	0.796
Post-treatment	1.98 ± 0.49	2.34 ± 0.53	−0.36 (−0.58 to −0.14)	0.001
Δ Change from baseline	−0.88 ± 0.66	−0.55 ± 0.84	−0.33 (−0.66 to −0.01)	0.043
RMSSD, ms				
Baseline	43.25 ± 9.20	42.90 ± 8.76	0.35 (−3.48–4.18)	0.856
Post-treatment	62.47 ± 10.46	53.18 ± 9.96	9.29 (4.93–13.65)	<0.001
Δ Change from baseline	19.22 ± 13.77	10.28 ± 14.89	8.94 (2.80–15.08)	0.005
pNN50, %				
Baseline	8.55 ± 4.12	8.30 ± 4.05	0.25 (−1.49–2.00)	0.775
Post-treatment	14.80 ± 5.67	11.27 ± 4.68	3.53 (1.32–5.73)	0.002
Δ Change from baseline	6.25 ± 7.80	2.97 ± 6.19	3.27 (0.29–6.26)	0.034

Values are presented as mean ± SD. Mean difference = observation group−control group. SDNN, standard deviation of all normal-to-normal intervals; RMSSD, root mean square of successive differences; LF/HF, low-frequency to high-frequency power ratio; pNN50, proportion of successive NN interval differences >50 ms.

### Secondary outcomes: cardiac function

**Figure 3:**
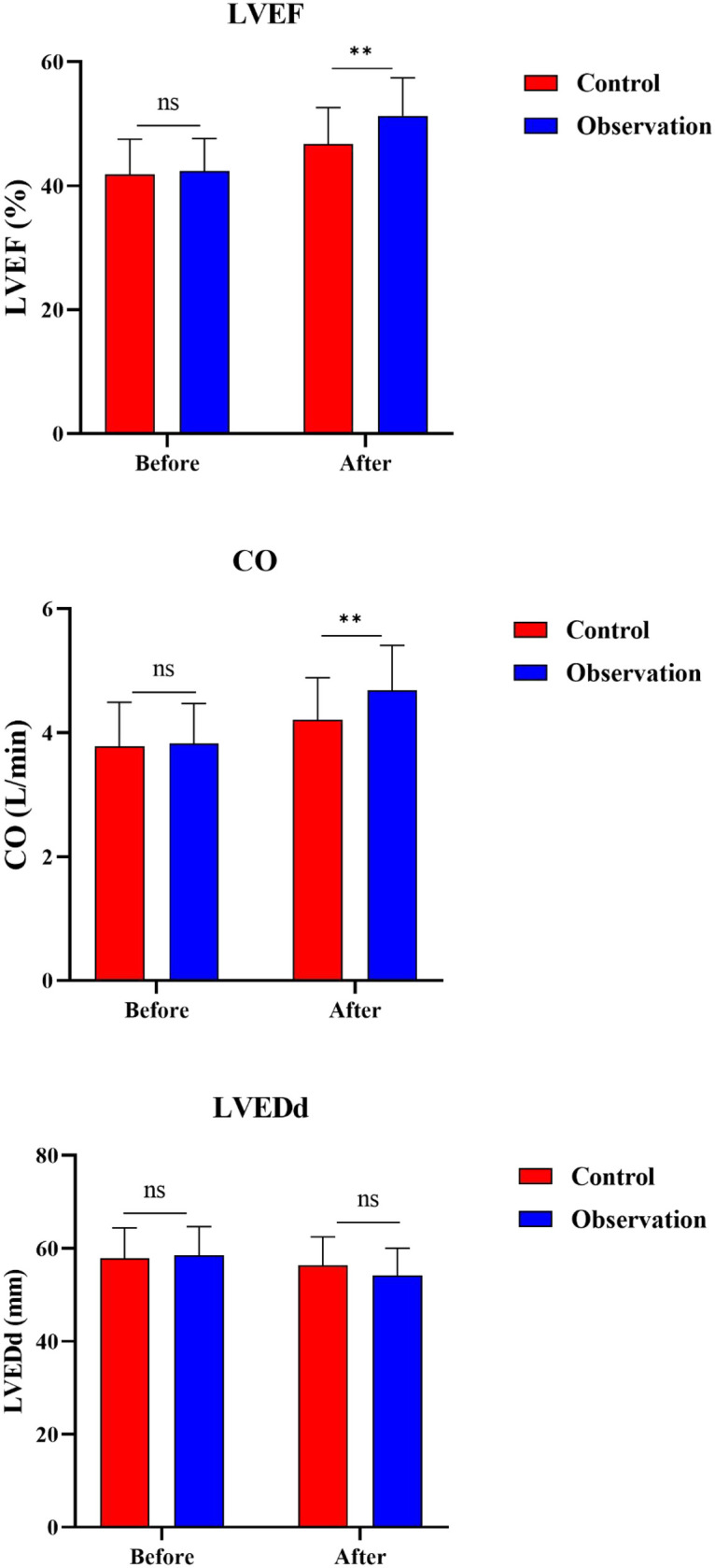
Changes in cardiac function parameters before and after treatment. **(A)** Left ventricular ejection fraction (LVEF); **(B)** Cardiac output (CO); **(C)** Left ventricular end-diastolic diameter (LVEDd). ** *P* < 0.001.

The comparison of cardiac function parameters are shown in Figure 3 and Table 3. Baseline measurements revealed no significant differences between the observation and control groups in LVEF (*P* = 0.680), CO (*P* = 0.783), or LVEDd (*P* = 0.692). The change in LVEF from baseline was 12.74 ± 8.56% in the observation group compared to 7.88 ± 8.11% in the control group [mean difference 4.86, 95% CI (1.31–8.41), *P* = 0.008]. The change in CO was also significantly greater in the observation group [ΔCO: 0.82 ± 0.95 vs. 0.32 ± 1.01 L/min; mean difference 0.50, 95% CI (0.08–0.92), *P* = 0.019]. The change in LVEDd from baseline did not differ significantly between groups [ΔLVEDd: −5.17 ± 9.86 vs. −3.33 ± 10.18 mm; mean difference −1.84, 95% CI (−6.12–2.45), *P* = 0.395].

**Table 3:** Comparison of cardiac function parameters before and after treatment.

Parameter	Observation group (*n* = 46)	Control group (*n* = 41)	Mean difference (95% CI)	*P*
LVEF, %				
Baseline	38.52 ± 5.86	38.85 ± 5.72	−0.33 (−2.80–2.14)	0.792
Post-treatment	51.26 ± 6.19	46.73 ± 5.96	4.53 (1.94–7.12)	<0.001
Δ Change from baseline	12.74 ± 8.56	7.88 ± 8.11	4.86 (1.31–8.41)	0.008
CO, L/min				
Baseline	3.86 ± 0.63	3.89 ± 0.61	−0.03 (−0.29–0.23)	0.823
Post-treatment	4.68 ± 0.74	4.21 ± 0.69	0.47 (0.17–0.77)	0.003
Δ Change from baseline	0.82 ± 0.95	0.32 ± 1.01	0.50 (0.08–0.92)	0.019
LVEDd, mm				
Baseline	59.35 ± 6.32	59.68 ± 6.48	−0.33 (−3.07–2.40)	0.810
Post-treatment	54.18 ± 5.93	56.35 ± 6.20	−2.17 (−4.77–0.42)	0.099
Δ Change from baseline	−5.17 ± 9.86	−3.33 ± 10.18	−1.84 (−6.12–2.45)	0.395

Values are presented as mean ± SD. Mean difference = observation group−control group. LVEF, left ventricular ejection fraction; CO, cardiac output; LVEDd, left ventricular end-diastolic diameter.

### Clinical efficacy and safety outcomes

The clinical efficacy and safety profiles of the two treatment regimens are summarized in Table 4. The overall response rate was numerically higher in the observation group (89.1%) compared to the control group (78.0%), but this difference did not reach statistical significance (*P* = 0.266).

**Table 4:** Clinical efficacy and safety outcomes.

Outcomes	Observation group (*n* = 46)	Control group (*n* = 41)	χ*^2^/t*	*P*
Clinical efficacy, *n* (%)				
Marked improvement	19 (41.3)	12 (29.3)		
Moderate improvement	22 (47.8)	20 (48.8)		
No improvement	5 (10.9)	9 (22.0)	2.540	0.281
Overall response rate, *n* (%)	41 (89.1)	32 (78.0)	1.236	0.266
Rehospitalization, *n* (%)	4 (8.7)	8 (19.5)	1.320	0.251
Mortality, *n* (%)	0 (0.0)	0 (0.0)	–	–
Adverse events, *n* (%)				
Total	5 (10.9)	4 (9.8)	–	1.000
Nausea	2 (4.3)	1 (2.4)		
Dizziness	2 (4.3)	2 (4.9)		
Bradycardia	1 (2.2)	0 (0.0)		
Fatigue	0 (0.0)	1 (2.4)		
Post-treatment resting HR, bpm	68.3 ± 8.6	72.6 ± 9.3	2.238	0.028

Values are presented as *n* (%) or mean ± SD. Overall response rate = (marked improvement + moderate improvement)/total × 100%.

The observation group exhibited a numerically lower rehospitalization rate (8.70%) compared to the control group (19.51%), though this difference did not reach statistical significance (*P* = 0.251). No deaths occurred in either group during the study period. Adverse reactions were infrequent and mild to moderate in severity in both groups, with no statistically significant difference in overall adverse reaction rates between the observation group (10.87%) and control group (9.76%). Post-treatment resting heart rate was significantly lower in the observation group than in the control group (68.3 ± 8.6 vs. 72.6 ± 9.3 bpm, *P* = 0.028).

### Sensitivity and adjusted analyses

To address potential confounding by AF-induced cardiomyopathy, a sensitivity analysis was performed excluding 23 patients (9 observation, 14 control) in whom AF was the primary HF etiology (Table 5). In this subgroup analysis, the between-group differences in ΔLVEF (*P* = 0.013) and ΔCO (*P* = 0.003) remained statistically significant. However, the differences in ΔSDNN (*P* = 0.123) and ΔRMSSD (*P* = 0.185) were no longer significant, while ΔLF/HF remained significant (*P* = 0.016).

**Table 5:** Sensitivity analysis excluding patients with AF as primary HF etiology.

Parameter	Observation group (*n* = 37)	Control group (*n* = 27)	Mean difference (95% CI)	*P*
Primary outcome: Δ HRV parameters				
ΔSDNN, ms	20.28 ± 23.25	11.21 ± 22.50	9.07 (−2.49–20.64)	0.123
ΔLF/HF	−0.84 ± 0.67	−0.40 ± 0.75	−0.44 (−0.81 to −0.08)	0.016
ΔRMSSD, ms	17.47 ± 13.94	12.41 ± 16.15	5.06 (−2.69–12.81)	0.185
Secondary outcome: Δ Cardiac function				
ΔLVEF, %	11.36 ± 8.41	6.22 ± 7.28	5.14 (1.20–9.08)	0.013
ΔCO, L/min	0.93 ± 0.99	0.15 ± 1.00	0.78 (0.27–1.28)	0.003
ΔLVEDd, mm	−4.68 ± 9.37	−3.26 ± 10.45	−1.42 (−6.51–3.66)	0.570
Clinical efficacy				
Overall response rate, *n* (%)	33 (89.2)	20 (74.1)		0.212

Sensitivity analysis excluding 23 patients (9 observation, 14 control) in whom atrial fibrillation was identified as the primary etiology of heart failure, to address the concern that LVEF improvements in these patients may reflect rate/rhythm control of AF-induced cardiomyopathy rather than a true drug effect. Values are presented as mean ± SD for Δ (change from baseline) or *n* (%).

Multivariable linear regression adjusting for age, sex, NYHA class, HF phenotype, baseline LVEF, NT-proBNP, AF etiology, and background GDMT use was performed to assess the robustness of the treatment effects (Table 6). After adjustment, the between-group differences in ΔSDNN [adjusted mean difference 11.59, 95% CI (0.71–22.46), *P* = 0.037] and ΔRMSSD [adjusted mean difference 7.33, 95% CI (0.27–14.39), *P* = 0.042] remained statistically significant. The adjusted differences in ΔLF/HF (*P* = 0.127), ΔLVEF (*P* = 0.062), and ΔCO (*P* = 0.091) did not reach statistical significance.

**Table 6:** Multivariable-adjusted treatment effects on primary and secondary outcomes.

Outcomes	Unadjusted mean difference	Adjusted mean difference (95% CI)^a^	*P*
ΔSDNN, ms	13.63	11.59 (0.71–22.46)	0.037
ΔRMSSD, ms	8.94	7.33 (0.27–14.39)	0.042
ΔLF/HF	−0.33	−0.29 (−0.66–0.08)	0.127
ΔLVEF, %	4.86	3.89 (−0.20–7.98)	0.062
ΔCO, L/min	0.50	0.41 (−0.07–0.90)	0.091
ΔLVEDd, mm	−1.84	−1.43 (−6.36–3.49)	0.568

a Adjusted for age, sex, NYHA functional class, HF phenotype (HFrEF vs. HFmrEF), baseline LVEF, NT-proBNP, AF as primary etiology, and background GDMT use (ACEi/ARB/ARNI, MRA, SGLT2i) using multivariable linear regression with treatment group as the independent variable. Dependent variable: change from baseline (Δ) for each outcome.

## Discussion

This retrospective study investigated whether adding bucladesine calcium to metoprolol provides additional autonomic and cardiac benefits in patients with HF and chronic arrhythmias. The combination group showed significantly greater improvements in ΔSDNN and ΔRMSSD, both remaining significant after multivariable adjustment. The between-group difference in ΔLF/HF ratio was borderline and non-significant after adjustment. Among secondary outcomes, ΔLVEF was greater in the combination group but attenuated after adjustment, while ΔLVEDd and overall clinical response rate did not differ significantly. Adverse event rates were comparable between groups.

Autonomic dysfunction represents a key role in the pathophysiology of HF, characterized by sympathetic overactivation and parasympathetic withdrawal (17, 18). HRV is markedly reduced in patients with HF, and the reduction correlates with disease severity and prognosis (7). The improvements in HRV observed in the combination group aligned with recent evidence establishing HRV as a crucial prognostic marker in HF populations. Studies have established reference values for HRV markers in HF and confirmed that values outside the reference range are significantly associated with mortality, particularly for frequency domain indices such as the LF/HF ratio (5). The LF/HF ratio has been shown to predict the development of HF in elderly cohorts, with its addition to traditional cardiovascular risk factors improving predictive performance by 6.1% (18). The magnitude of SDNN improvement observed in the combination group is clinically meaningful, as SDNN values below 40–121 ms are associated with poor prognosis in HFrEF patients. Recent systematic reviews further confirm HRV as a robust predictor of adverse outcomes in HF populations (7). However, when patients with AF as the primary HF etiology were excluded, ΔSDNN and ΔRMSSD lost statistical significance, though the effect direction was preserved and the reduced sample size likely limited statistical power. The combination group also demonstrated a significantly lower post-treatment resting heart rate (68.3 vs. 72.6 bpm), which may itself contribute to improved HRV indices independently of a direct autonomic effect.

The mechanisms underlying bucladesine calcium's cardiovascular effects involve cyclic AMP signaling. Cyclic nucleotides, particularly cAMP and cGMP, maintain physiological cardiac contractility, and in HF, alterations in multiple phosphodiesterases disrupt cyclic nucleotide levels and promote cardiac dysfunction (19). Bucladesine (dibutyryl cAMP) is a cell-permeable cAMP analog that directly activates protein kinase A (PKA), mechanistically distinct from selective PDE3 inhibitors such as milrinone that elevate endogenous cAMP by blocking its degradation (20). Through the cAMP–PKA axis, bucladesine modulates L-type Ca^2^⁺ channel activity, ryanodine receptor (RyR2) gating, and phospholamban phosphorylation, providing a plausible basis for the observed improvements in both contractile performance and autonomic indices (21). The safety of chronic cAMP pathway stimulation has been questioned since the PROMISE trial showed that oral milrinone, a selective PDE3 inhibitor, increased all-cause mortality by 28% and cardiovascular mortality by 34% in severe chronic HF (22). Bucladesine differs from milrinone in that it directly activates PKA as a cAMP analog rather than blocking PDE3-mediated cAMP degradation, thereby bypassing the compartment-specific cAMP accumulation implicated in PDE3i-related proarrhythmia (20). Additionally, bucladesine was administered intravenously for a defined 12-week course under clinical monitoring, unlike the chronic oral administration in the PROMISE trial. No excess arrhythmias or mortality were observed in our study, although the short follow-up and limited sample preclude definitive safety conclusions. Bucladesine is approved as a cardiotonic agent in Chinese practice under institutional protocols. no large-scale randomized trial has evaluated its long-term cardiovascular effects.

The LVEF improvement in the combination group, though attenuated after covariate adjustment, is consistent with the positive inotropic properties of cAMP signaling and the reverse-remodeling capacity of beta-blockers (23). The absence of a meaningful change in LVEDd suggests that 12 weeks was insufficient for structural remodeling, which typically requires 6–12 months of sustained therapy. Over one-quarter of patients had AF as the primary HF etiology, raising the possibility of tachycardia-induced cardiomyopathy in which LVEF recovery may reflect rate control rather than the study intervention (24). The persistence of the LVEF benefit after excluding these patients suggests that the cardiac function improvement was at least partially independent of rhythm management, though the small subgroup warrants caution. Both groups received metoprolol tartrate rather than the succinate formulation supported by MERIT-HF, reflecting local prescribing practice, and the comparable final doses argue against differential beta-blocker titration as an explanation for the observed effects.

The management of patients with concurrent arrhythmias and HF remains challenging. Different baseline sympathovagal balance and cardiac autonomic responsiveness exist between ischemic and non-ischemic HF (25), and the autonomic nervous system remains an underexplored therapeutic target despite the prognostic significance of autonomic imbalance (26). The combination regimen employed in this study tempt in this study attempted to address this gap by combining beta-adrenergic blockade with cAMP-mediated autonomic modulation.

This study has several limitations. The retrospective, non-randomized design introduces selection bias that cannot be fully addressed by multivariable adjustment; residual confounding may persist despite the robustness of the primary HRV findings after covariate correction. The sample size provided adequate power for the primary comparison but was insufficient for the AF-exclusion sensitivity analysis, likely explaining the loss of HRV significance in that subgroup, and the multivariable model approached the lower methodological boundary for covariate-to-sample ratio. Metoprolol tartrate was used rather than the succinate formulation with established mortality benefit in HF, and the target dose remained below international guideline recommendations; both reflect local prescribing practice but limit generalizability to other settings. The requirement for daily intravenous bucladesine over 12 weeks, feasible through outpatient infusion centers in Chinese hospitals, substantially restricts clinical applicability elsewhere. Finally, the 12-week follow-up was insufficient to assess hard endpoints such as mortality and major adverse cardiac events. Prospective, randomized, placebo-controlled trials using guideline-recommended beta-blocker formulations and incorporating hard clinical endpoints are warranted.

## Conclusion

Adding bucladesine calcium to metoprolol was associated with greater improvements in HRV time-domain parameters that persisted after multivariable adjustment, and a numerically larger LVEF improvement that was attenuated after covariate correction. Effects on sympathovagal balance and clinical response rate did not reach significance. These findings point to a potential role for cAMP-mediated autonomic modulation in HF with chronic arrhythmias, but require confirmation in prospective randomized trials.

## Data Availability

The original contributions presented in the study are included in the article/Supplementary Material, further inquiries can be directed to the corresponding author.
